# wTAM: a web server for annotation of weighted human microRNAs

**DOI:** 10.1093/bioadv/vbab040

**Published:** 2021-12-07

**Authors:** Chunmei Cui, Rui Fan, Yuan Zhou, Qinghua Cui

**Affiliations:** Department of Biomedical Informatics, MOE Key Lab of Cardiovascular Sciences, School of Basic Medical Sciences, Peking University, Beijing 100191, China

## Abstract

It is well-known that some microRNAs (miRNAs) are more important than the others for life, hinting the wide range of miRNA in essentiality or importance. Functional enrichment analysis is a quite pervasive method to dig out the underlying biological pathway for a given gene list and several tools of miRNA set enrichment analysis have been developed. However, all those tools treat each miRNA equally and neglect the importance score of miRNA itself, which could be an obstacle to seek more insightful biological processes for researchers. Here, we developed wTAM, a tool for annotation of weighted human miRNAs, introducing the miRNA importance scores into enrichment analysis. In addition, the annotation repository has been enlarged comparing to TAM. Finally, the case study demonstrated the availability and flexibility of wTAM.

**Availability and implementation:**

wTAM is freely available at http://www.cuilab.cn/wtam/.

**Supplementary information:**

Supplementary data are available at *Bioinformatics Advances* online.

## 1 Introduction

MicroRNAs (miRNAs) as a class of noncoding RNAs, regulating the expression or translation of messenger RNAs, play crucial roles in various biological processes and human diseases. The strategy to infer the potential pathways or functions that miRNAs involve in after screening differentially expressed miRNAs (DEMs) in a transcriptomic dataset is common and useful. Currently, there are several available tools for annotation of a given miRNA list (de Amorim *et al.*, 2022), e.g. TAM (Li *et al.*, 2018) and miEAA (Kern *et al.*, 2020). However, above methods treated each miRNA equally, i.e. all miRNAs are deemed to have the same importance, which is not in line with the real pathological contexts. Obviously, there are some particular miRNAs are more essential than the others for human. We previously devised a method to calculate miRNA importance score in the aspect of disease-association (Cui *et al.*, 2019), which predicted the effect of miRNAs on diseases. Therefore, it is necessary for introducing the miRNA weighting scores on enrichment analysis. A method about weighted enrichment analysis tool for genes, WEAT (Fan and Cui, 2021), has been presented, in which gene essentiality scores have been added in over-represented analysis of sets of gene of interest. It is still lack of relevant methods for miRNAs. Based on WEAT framework, we developed an online tool for annotation of weighted human miRNAs with over-represented analysis, called wTAM, and verified its availability with a case study.

## 2 The wTAM web server

### 2.1 Data collection

Firstly, we obtained 1239 well-annotated miRNA sets from TAM (Li *et al.*, 2018). We manually collected and annotated two new categories, cell specificity and environmental factor (EF). The cell expression specificity was calculated with the miRNA expression profile in 121 cell lines from the study of de Rie *et al.* (2017). The miRNA sets of EF-associated was based on the updated data of miREnvironment database (Yang *et al.*, 2011), which collected experimentally supported miRNAs and EF interplays. Up to now, there are 2475 miRNA sets covering 1764 miRNAs in wTAM, which largely enriches the miRNA annotation repository.

We already presented the MIC (Cui *et al.*, 2019) method to calculate the human miRNA importance score, which showed the difference in miRNA association with human diseases. Also, other features could mirror wide variety in the importance of miRNA itself. Herein, we compiled 5 categories and 17 subcategories for miRNA weighting scores, including MIC score, miRNA conservation score represented by the number of miRNA family members, miRNA expression score, downstream score, and upstream score of miRNA. The miRNA expression score in multiple tissues is from miRmine Database (Panwar *et al.*, 2017), where we only retained the normal samples and calculated the average of miRNA expression for multiple samples in each tissue. The details about miRNA weighting scores were shown in Supplementary Table S1.

### 2.2 Construction of wTAM

**Fig. 1.**
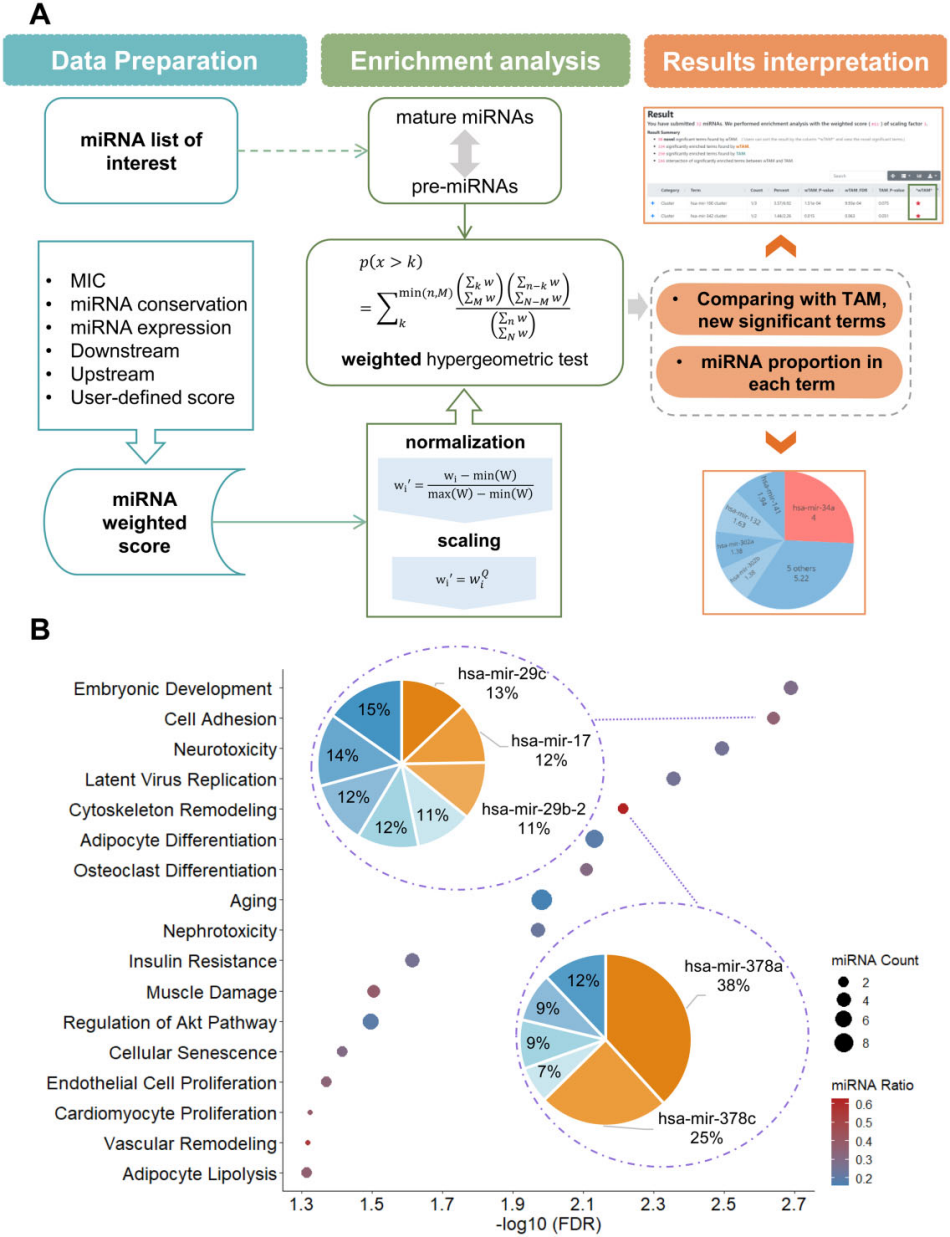
The construction and validation of wTAM. (**A**) The workflow of wTAM tool. (**B**) The new significant functions from wTAM comparing to TAM in case study. A total of 17 novel significant function terms were found by wTAM in 103 DEMs between ER+ and ER− patients. The size (miRNA count) indicates the number of intersection between a given miRNA set and input miRNA list. The color (miRNA ratio) shows the ratio of the weighting sum of hit miRNAs and all miRNA in a given miRNA set. Two pie charts show the weighting proportion of miRNAs in the set of ‘Cell adhesion’ and ‘Cytoskeleton remodeling’, respectively

Previous studies have shown the broad range of human miRNA importance/essentiality. The core of wTAM is absorbing the miRNA importance scores into miRNA set enrichment analysis. Similar to WEAT (Fan and Cui, 2021), the miRNA weighting score needs to be preprocessed, including normalization and scaling. In the conventional over-represented analysis, simple counts are believed as parameters into the hypergeometric test to examine whether each miRNA set is enriched in the input list. Here, we replaced counting with the sum of miRNA weighting scores in hypergeometric distribution, in which the calculation is based on real number, not limited on integers. The combination operation on nonintegral values could be implemented by Gamma function, generalization of the factorial function to nonintegral values. We completed it with the Special package in Python. The pipeline of wTAM was shown in Figure 1A.

## 3 Application for exploration new insightful biological processes

Breast cancer is a main threaten among the worldwide women, characterized by molecularly distinct tumor subtypes linking with different clinical outcomes (Gong *et al.*, 2019). To explore the practicability of wTAM, we acquired 103 DEMs between estrogen receptor negative (ER−, 29 patients) and ER+ (29 patients) subtype from Gong’s study (Gong *et al.*, 2019). We obtained the miRNA expression profile from TCGA-BRCA project, containing 1097 primary breast tumor tissue, and calculated the average of expression value in all samples for each miRNA. Subsequently, we performed enrichment analysis using wTAM with DEMs and the weighting score of miRNA expression in breast cancer as input. As a result, compared with TAM, 17 novel significant functions enriched in DEMs by wTAM were depicted in Figure 1B, of which Cell adhesion (FDR = 2.28E−3 in wTAM; FDR = 0.062 in TAM) and Cytoskeleton remodeling (FDR = 6.10E−3 in wTAM; FDR = 0.21 in TAM) are the crucial processes for breast cancer metastasis (Fridrichova and Zmetakova, 2019; Scully *et al.*, 2012), which suggested the distinct differences in tumor progression and invasion between ER− and ER+ subtype. The growth and proliferation of tumor cell are activated by the estrogen binding with estrogen receptor in ER+ subtype, while the occurrence of cancer for ER− subtype relies on other cell signaling pathways. Among the new significant pathways, we found that Regulation of Akt pathway is enriched in DEMs (FDR = 0.032 in wTAM; FDR = 0.088 in TAM), hinting the critical pathways of tumorigenesis for ER− breast cancer subtype. In addition, there exist some significant transcription factors regulating the transcription of DEMs, such as, TLR2, MYOD1 and REST, which all have relevance for the progression of breast cancer. All significant terms were shown in Supplementary Table S2. Comparing to conventional hypergeometric test, the contribution of each miRNA is not equal for a given miRNA set. We also exhibited the detailed proportion of miRNA weightings for above-mentioned miRNA sets, which might be helpful for exploring clinical therapy in different subtypes of breast cancer.

## 4 Conclusion

We developed a tool considering a priori weighting of miRNA on performing miRNA set enrichment analysis. We demonstrated wTAM could detect more insightful pathways than the conventional tool with a case study. Additionally, the miRNA annotation knowledge has been enlarged. Currently, with the limitation of miRNA sets organized by the pre-miRNAs and their related functions or phenotypes, wTAM only supported the enrichment analysis for pre-miRNAs. In the future, wTAM would contain two types of annotation information based on the pre-miRNAs and mature miRNAs, respectively. The user-friendly online tool would facilitate the research on disease mechanism and treatment.

## Funding

This work was supported by the grants from the National Key R&D Program (2020YFC2004704); PKU-Baidu Fund (2019BD014); the Natural Science Foundation of China (62025102/81970440/81921001); and Peking University Basic Research Program (BMU2020JC001).

## Data Availability

The data underlying this article are available in the article and in its online supplementary material.

*Conflict of Interest*: There is no conflict.

## Supplementary Material

vbab040_Supplementary_Data
