# Supplementary material for: wTAM: a web server for annotation of weighted human microRNAs
Source: Bioinform Adv. 2021 Dec 7;2(1):vbab040. doi: 10.1093/bioadv/vbab040 (PMC9710651; doi:10.1093/bioadv/vbab040)
Supplement: vbab040_Supplementary_Data [file vbab040_supplementary_data.zip › Supplementary-Table-S1.docx]

Supplementary Table S1. The detailed description and source of miRNA weighting scores.

| **Type** | **Description** | **Source** | **Ref.** |
| --- | --- | --- | --- |
| Conservation | The number of miRNA family members | miRBase | PMID: 30423142 |
| Expression | The miRNA expression level in 13 normal/control tissues | miRmine | PMID: 28108447 |
| MIC | miRNA importance score, which measures the association of miRNA and diseases | MIC | DOI: 10.1002/adts.201900083 |
| Downstream Regulation | The number of target genes regulated by miRNAs | miRTarBase | PMID: 29126174 |
| Upstream Regulation | The number of transcription factors regulating miRNAs | TransmiR | PMID: 30371815 |
